# Randomised phase I/II study to evaluate *c*arbon *i*o*n *ra*d*ioth*e*rapy ve*r*sus fractionat*e*d stereotactic radiotherapy in patients with recurrent or progressive g*l*iom*a*s: The CINDERELLA trial

**DOI:** 10.1186/1471-2407-10-533

**Published:** 2010-10-06

**Authors:** Stephanie E Combs, Iris Burkholder, Lutz Edler, Stefan Rieken, Daniel Habermehl, Oliver Jäkel, Thomas Haberer, Renate Haselmann, Andreas Unterberg, Wolfgang Wick, Jürgen Debus

**Affiliations:** 1 Department of Radiation Oncology, University Hospital of Heidelberg, Im Neuenheimer Feld 400, 69120 Heidelberg, Germany; 2 Institute of Medical Biometry and Informatics, University of Heidelberg, Im Neuenheimer Feld 305, 69120 Heidelberg, Germany; 3 Heidelberger Ionenstrahl Therapiezentrum (HIT), Im Neuenheimer Feld 450, 69120 Heidelberg, Germany; 4 Department of Neurooncology, University Hospital of Heidelberg, Im Neuenheimer Feld 400, 69120 Heidelberg, Germany; 5 Department of Neurosurgery, University Hospital of Heidelberg, Im Neuenheimer Feld 400, 69120 Heidelberg, Germany

## Abstract

**Background:**

Treatment of patients with recurrent glioma includes neurosurgical resection, chemotherapy, or radiation therapy. In most cases, a full course of radiotherapy has been applied after primary diagnosis, therefore application of re-irradiation has to be applied cauteously. With modern precision photon techniques such as fractionated stereotactic radiotherapy (FSRT), a second course of radiotherapy is safe and effective and leads to survival times of 22, 16 and 8 months for recurrent WHO grade II, III and IV gliomas.

Carbon ions offer physical and biological characteristics. Due to their inverted dose profile and the high local dose deposition within the Bragg peak precise dose application and sparing of normal tissue is possible. Moreover, in comparison to photons, carbon ions offer an increased relative biological effectiveness (RBE), which can be calculated between 2 and 5 depending on the GBM cell line as well as the endpoint analyzed. Protons, however, offer an RBE which is comparable to photons.

First Japanese Data on the evaluation of carbon ion radiation therapy for the treatment of primary high-grade gliomas showed promising results in a small and heterogeneous patient collective.

**Methods Design:**

In the current Phase I/II-CINDERELLA-trial re-irradiation using carbon ions will be compared to FSRT applied to the area of contrast enhancement representing high-grade tumor areas in patients with recurrent gliomas. Within the Phase I Part of the trial, the Recommended Dose (RD) of carbon ion radiotherapy will be determined in a dose escalation scheme. In the subsequent randomized Phase II part, the RD will be evaluated in the experimental arm, compared to the standard arm, FSRT with a total dose of 36 Gy in single doses of 2 Gy.

Primary endpoint of the Phase I part is toxicity. Primary endpoint of the randomized part II is survival after re-irradiation at 12 months, secondary endpoint is progression-free survival.

**Discussion:**

The Cinderella trial is the first study to evaluate carbon ion radiotherapy for recurrent gliomas, and to compare this treatment to photon FSRT in a randomized setting using an ion beam delivered by intensity modulated rasterscanning.

**Trial Registration:**

NCT01166308

## Background

Recurrent gliomas remain a major challenge in radiation oncology. In the past, second courses of radiotherapy have only been applied reluctantly, in fear of treatment-related side effects. However, modern radiation techniques have enabled the radiation oncologist to deliver high local doses as an effective salvage treatment with low rates of side effects.

Especially for GBM, with unsatisfactory outcomes even after extensive research over the past centuries, effective treatment at tumor recurrence are needed urgently. With surgery and supportive care alone, overall survival is about 3-5 months. Postoperative RT can increase overall survival to 9-12 months [1]. A number of studies have shown that an additional treatment with chemotherapy can increase overall survival. This benefit, however, is commonly associated with a high risk of treatment-related side effects, especially with combination treatments, such as PCV, and with BCNU. Only recently, significant increase in overall survival could be achieved by adding TMZ, an orally applicable alkylating substance, to postoperative radiotherapy. In a prospective randomized Phase III study performed by the EORTC radiochemotherapy with TMZ was compared to postoperative radiation alone. Overall survival could be increased from 12.1 months to 14.6 months, with acceptable toxicity. TMZ was applied in a dose of 75 mg/m^2^/die during radiotherapy, followed by 6 cycles of adjuvant TMZ [2]. Therefore, standard treatment of patients with GBM is currently considered to be postoperative radiochemotherapy with TMZ, followed by 6 cycles or adjuvant TMZ.

However, with an overall survival of about 15 months, treatment outcome still remains insufficient, and patients develop tumor recurrences soon after primary diagnosis.

For anaplastic gliomas, overall survival after standard treatment following primary diagnosis, including neurosurgical resection and postoperative radiotherapy, lies between 18 and 50 months [3]. However, also these patients develop tumor recurrences after primary diagnosis, and effective salvage treatments are required.

Treatment of low grade gliomas has been discussed controversially over the last years with respect to optimal radiation dose and time point of radiotherapy. It has been shown, that doses of 45-54 Gy are sufficient for long-term local tumor control, and that early application of radiotherapy can increase progression-free survival significantly, without altering overall survival as compared to treatment applied for tumor progression [4]. During follow-up, patients develop not only low grade tumor progression, but also high-grade malignant tumor lesions.

In the past, a second course of radiotherapy has been applied reluctantly with conventional techniques as treatment outcome outweighs the risk of treatment-related side effects [5]. With modern high-precision stereotactic photon techniques, such as Fractionated Stereotactic Radiotherapy (FSRT) re-irradiation could be established as a safe and effective treatment option for recurrent gliomas [6]. Survival times of 111, 50 and 21 months for WHO Grade II, III and IV gliomas could be shown with FSRT, with very low rates of side effects. However, outcome still remains unsatisfactory [7].

Charged particles provide the physical advantage of an inverted dose profile which enables steep dose gradients. Neighboring organs at risk and surrounding normal tissue can be spared from radiation doses. Additionally, heavy charged particles, such as carbon ions, as high-LET beams, are characterized by an enhanced RBE. For glioblastoma cell lines, RBE values between 2 and 5 have been reported depending on cell line and endpoint [8,9].

In general, GBM are treatment-resistant tumors. Early studies using a high-dose proton boost could show that total doses up to 90 Gy E were effective in preventing local tumor recurrences, however, such high doses were associated with high rates of side effects [10]. Therefore, in general, standard radiation therapy is applied up to a total dose of 60 Gy. Besides the physical advantages of high local dose deposition provided by protons, carbon ions are a promising treatment alternative due to their biological benefits. A clinical study performed by the National Institute of Radiological Sciences (NIRS) treated 48 patients with malignant gliomas with a carbon ion boost up to total doses of 24.8 Gy E, of which 32 were GBM [11]. In this study, no treatment related toxicity exceeding CTCAE Grade II or higher were observed. Therefore, the concept of a carbon ion radiotherapy for the treatment of recurrent gliomas with contrast enhancing lesions is a promising treatment alternative.

Carbon ion radiotherapy was available by the Department of Radiation Oncology at the Gesellschaft für Schwerionenforschung (GSI) in Darmstadt since 1997. Superior treatment results for a number of tumor entities, such as chordomas and chondrosarcomas of the skull base, as well as ACC have been shown, and carbon ion radiotherapy is currently performed in the clinical routine for these patients [12,13]. Safety of carbon ion radiotherapy with respect to critical organs at risk, such as the brain, brainstem or spinal chord, have been shown in these studies. At the Heidelberg Ion Therapy Center (HIT), treatment of over 1300 patients per year with Proton and Carbon ion RT is possible.

*In vitro *data for the treatment of GBM with carbon ions have shown superior effectivity compared to photons [14]. Our own data have shown a high RBE for carbon ion RT for GBM; additionally a combination of carbon ion radiotherapy and TMZ have been evaluated and show an additive effect in GBM-cell lines [8]. A first clinical study evaluating a carbon ion boost in patients with GBM was recently published by Mizoe et al. [15]. Median overall survival in patients with glioblastoma was 17 months; however, only small patient numbers were evaluated and standard chemotherapy with TMZ was not applied. In that study, the carbon ion boost was applied with increasing total doses up to 24.8 Gy E. While toxicity was low even in the high dose arm, the data showed that patients seem to benefit from the high dose carbon ion boost.

The promising results in primary GBM and the known physical and biological properties of carbon ion offer a promising treatment alternative that should be evaluated for the treatment of patients with recurrent gliomas.

In the present CINDERELLA trial, the impact of carbon ion radiotherapy using intensity modulated rasterscanning will be compared to FSRT which is considered the treatment standard alternative for patients with recurrent gliomas. In a first Phase I design, the RD of carbon ion radiotherapy for re-irradiation of gliomas will be determined. In the following Phase II part, the RD of carbon ions will be compared to standard photon radiotherapy delivered as FSRT,

## Methods and Design

### Study design

The trial will be performed as a single-center two-armed randomized Phase I/II study.

#### Phase I: Dose Escalation

Patients fulfilling the inclusion criteria will be treated with increasing total doses of carbon ion radiotherapy to RD of carbon ion radiotherapy for re-irradiation of recurrent gliomas.

Patients will be treated within seven increasing dose regimens starting at 10 × 3 GyE up to 16 × 3 GyE.

#### Phase II: Randomized Part

Patients fulfilling the inclusion criteria will be randomized into two arms:

Arm A - Experimental Arm

Carbon Ion Radiation Therapy

The total dose applied will be the RD determined in the Phase I part of the study protocol.

Arm B - Standard Arm

Fractionated Stereotactic Radiotherapy with Photons

Total Dose 36 Gy, 18 fractions, 2 Gy single dose

**Figure 1:**
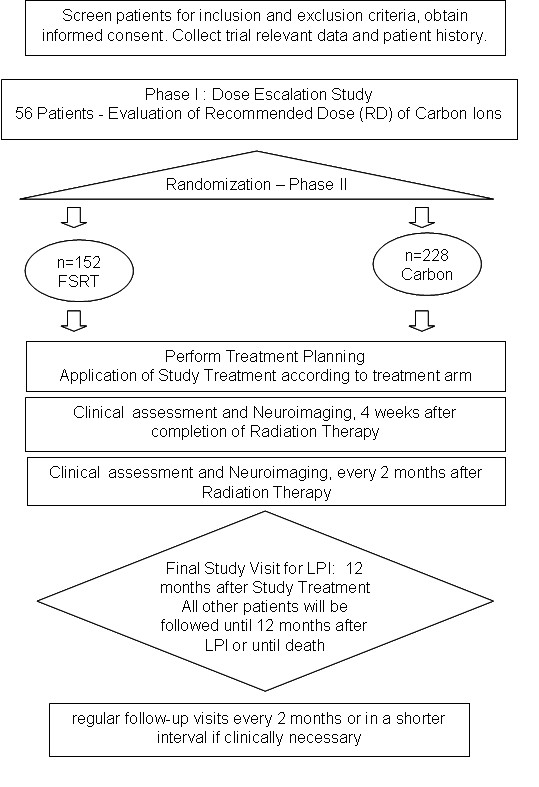
**Flow Chart of the Cinderella-Study**.

A flow chart of the study is shown in Fig. 1.

### Study objectives

The purpose of the trial is to compare carbon ion radiotherapy to FSRT for the treatment of recurrent gliomas delivered to the area of contrast-enhancement in T1-weighted MRI and/or Amino-Acid-PET-positive lesions.

In the Phase I study, the RD of carbon ion radiotherapy will be determined in a dose escalation scheme. The primary endpoint is toxicity.

In the randomized Phase II part of the study, carbon ion radiotherapy treated at the recommended dose evaluated in the phase I part will be compared to standard photon radiotherapy delivered as FSRT. The primary endpoint is survival after re-irradiation at 12 months.

### Primary Objectives

#### Phase I

The primary objective is any Grade IV toxicity related to the study treatment according to CTCAE Grade IV.

#### Phase II

The primary objective is survival after re-irradiation at 12 months.

### Secondary Objectives

#### Phase I

The secondary objective in the Phase I part is survival after re-irradiation

#### Phase II

The secondary objectives of the study are progression-free survival, toxicity and safety.

### Patient selection: Inclusion criteria

Patients meeting all of the following criteria will be considered for admission to the trial:

- unifocal, supratentorial recurrent glioma (primary histologies including any WHO Grade II or III glioma or glioblastoma)

- prior course of standard photon radiotherapy

- contrast enhancement on T1-weighted MRI and/or Amino-Acid-PET-positive high-grade tumor areas

- indication re-irradiation

- age ≥ 18 years of age

- Karnofsky Performance Score ≥60

- For women with childbearing potential, (and men) adequate contraception.

- Ability of subject to understand character and individual consequences of the clinical trial

- Written informed consent (must be available before enrolment in the trial)

### Patient selection: Exclusion criteria

Patients presenting with any of the following criteria will not be included in the trial:

- Multifocal Glioma or Gliomatosis cerebri

- refusal of the patients to take part in the study

- previous re-irradiation or prior radiosurgery or prior treatment with interstitial radioactive seeds

- time interval of < 6 months after primary radiotherapy

- Patients who have not yet recovered from acute toxicities of prior therapies

- Known carcinoma < 5 years ago (excluding Carcinoma in situ of the cervix, basal cell carcinoma, squamous cell carcinoma of the skin) requiring immediate treatment interfering with study therapy

- Pregnant or lactating women

- Participation in another clinical study or observation period of competing trials, respectively.

### Treatment Assignment

Radiation therapy according to the protocol will be performed in patients included into the study and after assignment of the patients to the treatment arms after randomization.

Patients withdrawn from the trial retain their identification codes (e.g. randomization number, if already given). New patients must always be allotted a new identification code.

### Treatment Planning

For FSRT and particle therapy, patients will be immobilized using an individually manufactured head mask. For treatment planning, contrast-enhanced CT as well as MR-imaging will be performed for optimal target definition.

Treatment planning for carbon ion and FSRT is to be performed about 1-2 weeks prior to the start of re-irradiation.

Organs at risk such as the brain stem, optic nerves, chiasm and spinal chord will be contoured. Dose constraints of normal tissue will be respected according to Emami et al. [16].

The Treatment Volume for Re-Irradiation will be defined as the area of contrast enhancement on T1-weighted MR-imaging adding a safety margin of 5 mm.

Amino-Acid-PET or SPECT-Examinations may be used in addition to contrast-enhanced MRI for target volume definition but are not mandatory.

FSRT planning is performed using standard photon-treatment planning systems such as HELAX, Masterplan or STP.

Carbon ion RT planning is performed using the treatment planning software PT-Planning (Siemens, Erlangen, Germany) including biologic plan optimization. Biologically effective dose distributions will be calculated using the a/β ratio for gliomas as well as for the endpoint late toxicity to the brain.

No interruptions > 4 days during study treatment (carbon ion radiotherapy or FSRT) are allowed.

Patient positioning prior to FSRT or particle therapy will be evaluated by comparison of x-rays to the DRRs. Set up deviations >3 mm are corrected prior to radiotherapy.

### Dose Prescription Experimental (Carbon) Arm

The intensity-controlled rasterscan system will be used for beam application. In the Phase I Dose Escalation Part, increasing doses of carbon ion radiotherapy will be evaluated in 7 steps starting at 10 × 3 Gy E up to 16 × 3 Gy E. In this part, the RD of carbon ion radiotherapy for re-irradiation of gliomas will be determined.

Therafter, the RD determined will be chosen for the randomized phase II part of the study. The total dose will be prescribed to the maximum of the calculated dose distribution for the target volume. Treatment planning aims in the coverage of the target volume by the 90%-isodose line.

Dose specification is based on biologic equivalent dose because of the high RBE of carbon ions, which differs throughout the target volume due to its dependence on various factors. RBE will be calculated at each voxel throughout the target volumes and biological optimization will be performed. The dose prescription used is related to the isoeffective dose GyE using daily fractions of 2 Gy and a weekly fractionation of 5 × 3 Gy.

**Figure 2:**
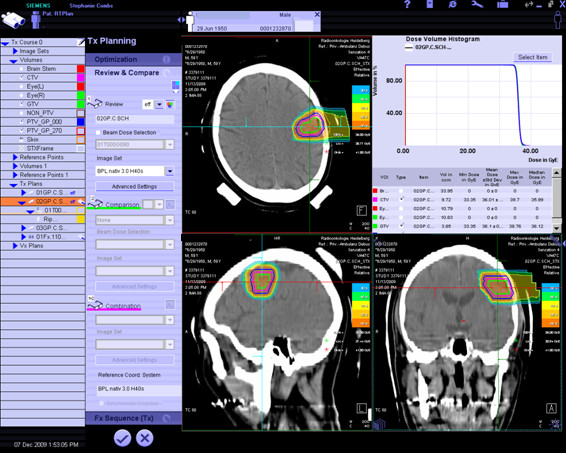
**Typical treatment plan for carbon ion radiotherapy for a patients with a recurrent glioblatoma**. Treatment is performed using the Siemens TPS for particle therapy, target volume delienation is based on CT- and MR-imaging with contrast enhancement.

A typical treatment plan is shown in Fig. 2.

### Dose Prescription Standard (Photon) Arm

Eighteen fractions of a single dose of 2 Gy up to a total dose of 36 Gy will be prescribed to the isocenter. Treatment planning aims in the coverage of the target volume by the 90%-isodose line.

### Statistical calculations for trial sample size

The statistical methods applied for this study are subject to GCP guidelines (Guidelines of the International Conference on Harmonisation (ICH) e.g.

• ICH E3: Structure and Contents of Clinical Study Reports,

• ICH E6: Good Clinical Practice (GCP). Consolidated Guideline,

• ICH E9: Note for Guidance on Statistical Principles in Clinical Trials) and will be performed in accordance with CESAR SOP 8 (Statistical Analysis/Biometry) in their versions valid at the date of the original study protocol.

### Study Hypothesis

#### Phase I

Phase I part of this study is conducted to choose the Recommended Dose (RD) of carbon ion radiotherapy for the phase II part between seven dose levels based on the dose escalation scheme.

#### Phase II

The phase II part of this study is designed to demonstrate superiority in survival of carbon ion radiotherapy (experimental) to FSRT (standard) in patients with recurrent or progressive gliomas. The primary endpoints variable is overall survival time after at least 12 months of follow-up (OS_12m) defined as time to death for any reason during the follow-up period of at least 12 months starting from date of randomization.

### Sample Size Calculation

#### Phase I

Seven DLs are included in the dose finding part. It is assumed that the probability of a DLT is 1/3. With n = 8 patients in each DL, the power that at each DL at least one DLT is observed is greater than 95%. Therefore a maximum of 56 patients will be treated in the phase I part of this study.

Within this study, DLTs are defined as any grade IV toxicity according to CTCAE Version 4.0 associated to study treatment.

#### Phase II

• The size of the phase II part of this study is determined by the primary aim of testing experimental treatment for superiority over standard treatment. This endpoint is a censored failure time.

• Information on the OS_B__12mR (overall survival rate after 12 months of follow-up for standard treatment) is given in literature data [17].

To calculate survival data, a population distribution of GBM in 34.3%, Grade III Tumors in 24.4% and low grade gliomas in 41.3% was used [17]. Survival rates for overall survival were taken as published previously with 23% for GBM 65% for Grade III, and 77% for low-grade gliomas observed at 12 months after re-irradiation [17].

Overall OS_B__12mR can be calculated as weighted mean rate. Therefore OS_B__12mR is expected to be 56%. The study will be designed to detect an improvement in OS_12mR of 10% (OS_A__12mR = 66%).

The statistical error rates for this test of superiority are set as α = 0.05 and β = 0.20 (power 80%). Sample size calculation is based on an unstratified log-rank. It can be expected that including histology in a stratified log-rank test will increase the power as compared to the unstratified test.

The recruitment period will be 36 months with a minimal follow-up time of 12 months. In order to put more patients on the experimental treatment, an unbalanced randomization allocation ratio of 2:3 was chosen.

For this set of assumptions, the sample size calculation programme PASS 2008 yields the total sample size for standard treatment of n = 138 and n = 207 for experimental treatment. Allowing for failures for various reasons of 10%, a total of N = 380 is required for this part of the study (n = 152 in standard arm and n = 228 in experimental arm). Patients will be stratified by histology.

### Statistical Methods

#### Phase I

##### Primary endpoint

Safety will be assessed by the type, incidence and severity (graded by the NCI CTC-AE Version 4.0) and relation to treatment. All patients treated at least once with one of the seven dose levels defined above will be described individually and summarized by dose level. No confirmatory statistical analysis will be performed.

### Secondary endpoint

Descriptive summary tables will be presented on baseline patient characteristics as well as for all safety parameters by dose level. Patients will be monitored for adverse events using NCI-CTC version 4.0.

#### Phase II

##### Primary endpoint

The primary endpoint variable OS_12 m was planned to be analysed by a one sided logrank test of stratified by histology testing H_0_: OS_A_-12 m = OS_B_-12 m versus H_1_: OS_A_-12 m > OS_B_-12 m at the level of alpha = 5%. One interim analysis is planned when 50% of the number of expected events under the null-hypothesis have occurred. In order to preserve the significance level of 5% a sequential plan with alpha-spending of De-Mets and Lan with limits of O'Brien and Fleming is applied.

Overall survival will be summarized by Kaplan-Meier curves. Median estimates as well as associated 95% confidence intervals will be reported. Further, a descriptive analysis of the primary outcome variable is performed applying a Cox-regression model.

### Secondary endpoints

• Progression-free survival will be analyzed analogously to the primary endpoints. P-value of the logrank test and test within the Cox regression will be interpreted descriptively.

• Safety/Toxicity will be assessed by the type, incidence, severity (graded by the NCT CTCAE Version 4.0), and relatedness of AEs to treatment and by assessment of laboratory parameters related to safety. Tolerability and dosing will be described by numbers of patients in whom treatment was given as planned, delayed or permanently stopped. All analyses will be performed separately for both treatment groups.

A detailed SAP will be designed after initiating the study and before start of data analysis by the biometric center. It will consider all analyses necessary to characterize the study populations and to evaluate the primary and secondary endpoints.

### Interim Analysis

#### Phase I

Besides the planned analysis during and after phase I part, no further interim analysis are planned.

#### Phase II

For the primary aim and the primary endpoint of the phase II, one interim analysis will be performed when 40% of the expected events under the null-hypothesis have occurred. Under the study hypotheses, 114 events will be occurred under the null hypothesis approximately after 26 months after start of recruitment.

Using O'Brien-Fleming approach with one scheduled interim analysis, hypothesis testing would be conducted at an interim significance level of α = 0.008. If significance is found in favour of the test treatment, the study may be stopped with sufficient statistical evidence of efficacy. If the level of significance is not reached at the interim analysis, the study continues to normal completion at which time the hypothesis testing is conducted at a significance level of α = 0.042. This stagewise procedure will have an overall significance of α = 0.05.

If for any reason no confirmative interim analysis will be performed, the final significance level will be 5%.

### Dose Escalation Scheme

The dose-escalation part of this study was designed to enroll successive cohorts of 8 patients, each to be started on a fixed dose of carbon ion therapy. The starting dose was specified as 10 × 3 Gy E for the first cohort. Planned dose level for subsequent cohorts were 11 × 3 Gy E, 12 × 3 Gy E, 13 × 3 Gy E, 14 × 3 Gy E, 15 × 3 Gy E and 16 × 3 Gy E.

Dose escalation was to be stopped when the maximum tolerated dose (MTD) was reached. MTD was defined as one dose level below that at which dose limiting toxicities (DLT) was observed in one-third or more of the patients meaning that at least 3 of the 8 patients in one dose cohort experienced a DLT. If maximum 2 DLTs were observed at one dose level, the next higher dose level will be evaluated. The maximum tolerated dose corresponds to the recommended dose for the following phase II part of this trial.

Toxicities were graded using the NCI Common Toxicity Criteria Version 4.0. DLT was defined as any treatment-related event qualifying as NCI grade 4.

### Ethics, informed consent and safety

A positive Ethics Vote was obtained by the Local Ethics Committee of the medical Faculty at the University of Heidelberg, Germany.

Before study recruitment, a positive vote of the Bundesamt für Strahlenschutz (BfS) is necessary.

### Treatment at tumor progression

After completion of study treatment not adjuvant treatment is conducted as part of this protocol. Further treatments may be initiated as needed clinically.

For tumor progression, treatment alternatives will be evaluated and discussed in the interdisciplinary setting considering options of neurosurgical resection, systemic treatment such as chemotherapy, a second course of radiation therapy, or other.

## Discussion

Treatment of recurrent gliomas still remains an interdisciplinary challenge. Re-irradiation using high-precision photon techniques has been established as a standard treatment in this clinical situation for a subgroup of patients: Several different fractionation schemes are applied in this setting, including hypofractionated regimens or radiosurgery, however, in our institution, Fractionated Stereotactic Radiotherapy (FSRT) with an attempted total dose of 36 Gy in 2 Gy single fractions is considered standard treatment [18-23]. Still outcome of these patients is not satisfactory and novel treatment appraoches are required.

With heavy charged particle beams such as carbon ions, the physical benefits of an ion beam in conjunction with the higher relative biological effectiveness (RBE) can be exploited for the treatment of different tumor entities. For several indications, superior clinical results as compared to photon radiotherapy have been shown [24]. Preclinical studies have shown the RBE to be between 3 and 5 for high-grade gliomas, therefore demonstrating that carbon ions are a promising treatment alternative for patients with recurrent gliomas [8,25].

Early clincial data on carbon ion radiotherapy in primary high-grade brain tumors have shown overall safety even with extensive dose escalation with promising results; however, few patients had been included into this study, and therefore validation in a larger patients collective is warranted [26].

Until now, no patients have been treated with carbon ion radiotherapy for recurrent gliomas. Based on the preclinical data as well as the clinical experience so far, this concept should be evaluated.

Therefore, the present CINDERELLA trial evaluates carbon ion radiotherapy performed as re-irradiation in patients with recurrent gliomas compared to FSRT with standard dosing. In a first Phase I part of the study, a dose escalation will be performed to determine the optimal dose of carbon ion radiotherapy that can be prescribed in this clinical setting. Thereafter, in the Phase II part of the trial, patients will be randomized between carbon ion radiotherapy and FSRT.

## Abbreviations

ACC: Adenoid Cystic Carcinomas; BCNU: Carmustine; DL: Dose level; DLT: Dose limiting toxicity; FSRT: Fractionated Stereotactic Radiotherapy; GBM: Glioblastoma; GSI: Gesellschaft für Schwerionenforschung; GY E: Gray Equivalent; HIT: Heidelberger Ionenstrahl herapiezentrum; NIRS: National Institute for Radiological Sciences; RBE: Relative Biological Effectiveness; RD: Recommended Dose; RT: Radiotherapy; TMZ: Temozolomide; WHO: World Health Organization

## Competing interests

The authors declare that they have no competing interests.

## Authors' contributions

SEC, JD, IB, LE and WW have developed the study concept. SEC, JD, IB and LE wrote the study protocol and obtained ethics approval. SEC, JD, WW, AU, SR and DH will provide patient care. TH and OJ will perform treatment planning and beam application for carbon ion radiotherapy. SEC, WW and JD will implement the protocol and oversee collection of the data. All authors contributed to and approved the final manuscript.

## Pre-publication history

The pre-publication history for this paper can be accessed here:

http://www.biomedcentral.com/1471-2407/10/533/prepub

## References

[B1] Walker MD Strike TA Sheline GE An analysis of dose-effect relationship in the radiotherapy of malignant gliomas Int J Radiat Oncol Biol Phys 1979 5 1725 31 231022 10.1016/0360-3016(79)90553-4

[B2] Stupp R Mason WP van den Bent MJ Weller M Fisher B Taphoorn MJ Radiotherapy plus concomitant and adjuvant temozolomide for glioblastoma N Engl J Med 2005 352 987 96 10.1056/NEJMoa043330 15758009

[B3] Nagy M Schulz-Ertner D Bischof M Welzel T Hof H Debus J Long-term outcome of postoperative irradiation in patients with newly diagnosed WHO grade III anaplastic gliomas Tumori 2009 95 317 24 19688970 10.1177/030089160909500308

[B4] van den Bent MJ Afra D de Witte O Ben Hassel M Schraub S Hoang-Xuan K Long-term efficacy of early versus delayed radiotherapy for low-grade astrocytoma and oligodendroglioma in adults: the EORTC 22845 randomised trial Lancet 2005 366 985 90 10.1016/S0140-6736(05)67070-5 16168780

[B5] Bauman GS Sneed PK Wara WM Stalpers LJ Chang SM McDermott MW Reirradiation of primary CNS tumors Int J Radiat Oncol Biol Phys 1996 36 433 41 8892469 10.1016/s0360-3016(96)00315-x

[B6] Combs SE Thilmann C Edler L Debus J Schulz-Ertner D Efficacy of fractionated stereotactic reirradiation in recurrent gliomas: long-term results in 172 patients treated in a single institution J Clin Oncol 2005 23 8863 9 10.1200/JCO.2005.03.4157 16314646

[B7] Combs SE Thilmann C Edler L Debus J Schulz-Ertner D Efficacy of fractionated stereotactic reirradiation in recurrent gliomas: long-term results in 172 patients treated in a single institution J Clin Oncol 2005 23 8863 9 10.1200/JCO.2005.03.4157 16314646

[B8] Combs SE Bohl J Elsaesser T Weber KJ Schulz-Ertner D Debus J Radiobiological evaluation and correlation with the local effect model (LEM) of carbon ion radiation therapy and temozolomide in glioblastoma cell lines Int J Rad Biol 2008 in press 10.1080/09553000802641151 19280465

[B9] Iwadate Y Mizoe J Osaka Y Yamaura A Tsujii H High linear energy transfer carbon radiation effectively kills cultured glioma cells with either mutant or wild-type p53 Int J Radiat Oncol Biol Phys 2001 50 803 8 11395250 10.1016/s0360-3016(01)01514-0

[B10] Fitzek MM Thornton AF Rabinov JD Lev MH Pardo FS Munzenrider JE Accelerated fractionated proton/photon irradiation to 90 cobalt gray equivalent for glioblastoma multiforme: results of a phase II prospective trial J Neurosurg 1999 91 251 60 10.3171/jns.1999.91.2.0251 10433313

[B11] Mizoe JE Tsujii H Hasegawa A Yanagi T Takagi R Kamada T Phase I/II clinical trial of carbon ion radiotherapy for malignant gliomas: combined X-ray radiotherapy, chemotherapy, and carbon ion radiotherapy Int J Radiat Oncol Biol Phys 2007 69 390 6 17459607 10.1016/j.ijrobp.2007.03.003

[B12] Schulz-Ertner D Karger CP Feuerhake A Nikoghosyan A Combs SE Jakel O Effectiveness of carbon ion radiotherapy in the treatment of skull-base chordomas Int J Radiat Oncol Biol Phys 2007 68 449 57 17363188 10.1016/j.ijrobp.2006.12.059

[B13] Schulz-Ertner D Nikoghosyan A Hof H Didinger B Combs SE Jakel O Carbon ion radiotherapy of skull base chondrosarcomas Int J Radiat Oncol Biol Phys 2007 67 171 7 17056193 10.1016/j.ijrobp.2006.08.027

[B14] Iwadate Y Mizoe J Osaka Y Yamaura A Tsujii H High linear energy transfer carbon radiation effectively kills cultured glioma cells with either mutant or wild-type p53 Int J Radiat Oncol Biol Phys 2001 50 803 8 11395250 10.1016/s0360-3016(01)01514-0

[B15] Mizoe JE Tsujii H Hasegawa A Yanagi T Takagi R Kamada T Phase I/II clinical trial of carbon ion radiotherapy for malignant gliomas: combined X-ray radiotherapy, chemotherapy, and carbon ion radiotherapy Int J Radiat Oncol Biol Phys 2007 69 390 6 17459607 10.1016/j.ijrobp.2007.03.003

[B16] Emami B Lyman J Brown A Coia L Goitein M Munzenrider JE Tolerance of normal tissue to therapeutic irradiation Int J Radiat Oncol Biol Phys 1991 21 109 22 2032882 10.1016/0360-3016(91)90171-y

[B17] Combs SE Thilmann C Edler L Debus J Schulz-Ertner DL Efficacy of fractionated stereotactic reirradiation in recurrent gliomas: long-term results in 172 patients treated in a single institution J Clin Oncol 2005 23 8863 9 10.1200/JCO.2005.03.4157 16314646

[B18] Combs SE Gutwein S Thilmann C Debus J Schulz-Ertner D Reirradiation of recurrent WHO grade III astrocytomas using fractionated stereotactic radiotherapy (FSRT) Strahlenther Onkol 2005 181 768 73 10.1007/s00066-005-1415-6 16362786

[B19] Combs SE Thilmann C Edler L Debus J Schulz-Ertner D Efficacy of fractionated stereotactic reirradiation in recurrent gliomas: long-term results in 172 patients treated in a single institution J Clin Oncol 2005 23 8863 9 10.1200/JCO.2005.03.4157 16314646

[B20] Combs SE Widmer V Thilmann C Hof H Debus J Schulz-Ertner D Stereotactic radiosurgery (SRS): treatment option for recurrent glioblastoma multiforme (GBM) Cancer 2005 104 2168 73 10.1002/cncr.21429 16220556

[B21] Combs SE Gutwein S Thilmann C Huber P Debus J Schulz-Ertner D Stereotactically guided fractionated re-irradiation in recurrent glioblastoma multiforme J Neurooncol 2005 74 167 71 10.1007/s11060-004-2463-y 16193388

[B22] Combs SE Ahmadi R Schulz-Ertner D Thilmann C Debus J Recurrent low-grade gliomas: the role of fractionated stereotactic re-irradiation J Neurooncol 2005 71 319 23 10.1007/s11060-004-2029-z 15735924

[B23] Combs SE Debus J Schulz-Ertner D Radiotherapeutic alternatives for previously irradiated recurrent gliomas BMC Cancer 2007 7 167 10.1186/1471-2407-7-167 17760992 PMC2212655

[B24] Schulz-Ertner D Tsujii H Particle radiation therapy using proton and heavier ion beams J Clin Oncol 2007 25 953 64 10.1200/JCO.2006.09.7816 17350944

[B25] Iwadate Y Mizoe J Osaka Y Yamaura A Tsujii H High linear energy transfer carbon radiation effectively kills cultured glioma cells with either mutant or wild-type p53 Int J Radiat Oncol Biol Phys 2001 50 803 8 11395250 10.1016/s0360-3016(01)01514-0

[B26] Mizoe JE Tsujii H Hasegawa A Yanagi T Takagi R Kamada T Phase I/II clinical trial of carbon ion radiotherapy for malignant gliomas: combined X-ray radiotherapy, chemotherapy, and carbon ion radiotherapy Int J Radiat Oncol Biol Phys 2007 69 390 6 17459607 10.1016/j.ijrobp.2007.03.003

